# Dietary Fatty Acids and Metabolic Health

**DOI:** 10.3390/nu17152512

**Published:** 2025-07-31

**Authors:** Éva Szabó

**Affiliations:** Department of Biochemistry and Medical Chemistry, Medical School, University of Pécs, 7624 Pécs, Hungary; szabo.eva.dr@pte.hu

Fatty acids are essential in human physiology, serving as primary energy sources, forming membrane lipids, and acting as cellular signaling molecules, thereby playing a significant role in the etiology of metabolic syndrome [1]. They are also crucial to the development and progression of various metabolic disorders, such as insulin resistance and cardiovascular diseases [2]. This Special Issue compiles publications that explore the role of dietary fatty acids in metabolic health through a multidimensional approach.

Excessive intake of dietary fats, particularly saturated fats, is strongly associated with an increased risk of chronic diseases such as obesity, diabetes, cardiovascular diseases, and hepatic metabolic disorders [3,4]. High-fat diets (HFD) can lead to obesity, hyperlipidemia, and metabolic disorders by altering the gut microbiota composition, reducing microbial diversity, and increasing the Firmicutes-to-Bacteroidetes ratio [4]. These changes in gut microbiota can enhance intestinal permeability, exacerbate inflammatory responses, and disrupt metabolic functions [4].

Dietary fatty acids have diverse and often opposing effects on blood lipid levels and the pathogenesis of cardiovascular diseases (CVDs). For instance, saturated fatty acids (SFAs) and *trans* isomeric fatty acids (TFAs) are linked to the development of atherosclerosis, while monounsaturated fatty acids (MUFAs) as well as *n*-3 polyunsaturated fatty acids (PUFAs) may offer cardioprotective benefits. However, research also suggests that SFAs and TFAs should not be uniformly considered as having solely adverse effects. Randomized clinical trials have shown that, despite coconut oil’s high content of medium-chain SFAs, it may not negatively impact blood lipids in healthy individuals. In these studies, short-term supplementation resulted in increased high-density lipoprotein (HDL) cholesterol levels, although the effect on low-density lipoprotein (LDL) cholesterol levels was inconsistent [5,6]. A meta-analysis further supported that medium-chain SFAs can raise HDL cholesterol levels without affecting triglyceride (TG) or total cholesterol levels [7].

TFAs can be divided into two subgroups: industrial and ruminant-origin TFAs. While the consumption of TFAs is generally linked to an increased risk of dyslipidemia and cardiovascular diseases, this risk is primarily associated with industrial-origin TFAs (iTFAs), whereas the effects of ruminant-origin TFAs (rTFAs) remain a topic of debate [8,9]. Evidence suggests that both total TFA and iTFA intakes are connected to a heightened risk of coronary heart disease (CHD) and CHD mortality, whereas rTFA intake seems to have no significant impact [10]. Conversely, recent findings indicate that both forms of TFAs may elevate cardiovascular risk factors, although rTFAs might be comparatively less harmful [11].

On the other hand, *n*-3 PUFAs are generally recognized for their beneficial effects, but not all members of this family have the same impact. In the STRENGTH trial, a daily supplementation of 4 g of eicosapentaenoic acid (EPA) and docosahexaenoic acid (DHA) did not affect the incidence of major adverse cardiovascular events, including cardiovascular death, nonfatal myocardial infarction, and stroke [12]. In contrast, the REDUCE-IT trial found that a 4 g/day EPA ethyl ester supplementation can significantly reduce the hazard ratio of cardiovascular death, stroke, and myocardial infarction [13]. A recent meta-analysis showed that while there was little or no effect of higher *n*-3 long-chain PUFA intake on the risk of cardiovascular events or mortality, it could reduce serum TG levels by 15% [14]. Additionally, a dose–response meta-analysis demonstrated a strong TG and non-HDL-lowering effect of EPA + DHA supplementation [15].

This Special Issue presents a collection of seven original research papers and a review that highlights the importance of dietary fatty acids in various aspects of metabolic diseases. A HFD is known to induce obesity, and in animal studies, it serves as a tool to explore the diverse aspects of obesity-related metabolic disorders. In a rat study, both dietary peanut oil and high-oleic peanut oil were found to enhance the diversity of intestinal microbiota and improve dyslipidemia and insulin sensitivity when compared to saturated fats in animals fed a high-fat/high-fructose diet (contribution 1). Additionally, a HFD is utilized to study metabolic dysfunction-associated fatty liver disease (MASLD) in animal models. Camellia seed cake extract, which is rich in several bioactive components, has been shown to reduce body weight and serum levels of total cholesterol, TG, and LDL-cholesterol. Furthermore, it decreased hepatic lipid accumulation, mitigated oxidative stress, and upregulated the expression of genes involved in coenzyme Q biosynthesis, thereby increasing coenzyme Q levels (contribution 4). Maternal obesity during pregnancy affects not only maternal but also offspring health, leading to complications during pregnancy, delivery, and later in life. A maternal HFD increased the weight of adipose tissue in the offspring, altered the fatty acid composition of their organs, and resulted in both metabolic and behavioral disturbances, such as anxiety-like behavior (contribution 5). Moreover, a maternal HFD during pregnancy can cause morphological changes in the offspring’s brain, impacting normal brain development. This effect could be mitigated with maternal fish oil supplementation, which is rich in EPA and DHA (contribution 6).

A high-quality diet and adherence to healthy eating patterns are associated with a reduced prevalence of metabolic syndrome and other cardiometabolic biomarkers. An American study demonstrated that a higher healthy eating index score correlates with a lower body mass index and a decreased incidence of metabolic syndrome. Additionally, several lipid species were found to be associated with a higher healthy eating index score, which in turn were strongly linked to improved cardiometabolic indicators (contribution 2). Dietary interventions have proven to be effective strategies for influencing cardiometabolic risk factors. Among African Americans, a reduction in dietary SFAs resulted in increased lipoprotein(a) levels across apolipoprotein(a) size, and in certain instances, led to a reclassification of cardiovascular risk (contribution 3). While *n*-3 PUFAs confer several health benefits, the underlying mechanisms remain not fully elucidated. A systematic review consolidates current knowledge on the modulation of metabolic disease pathways by dietary omega-3 PUFAs, drawing on findings from various omics studies, including lipidomics, proteomics, transcriptomics, and metabolomics (contribution 7). TFAs are known to have several adverse effects on human health; however, their role in type 1 diabetes mellitus remains unclear. In young diabetic adults and children, lower blood TFA values were observed. Yet, in cases of diabetic ketoacidosis, TFA values were higher compared to healthy peers or diabetic children without diabetic ketoacidosis (contribution 8).

## Abbreviations

The following abbreviations are used in this manuscript:

CHD	coronary heart disease
CVD	cardiovascular disease
DHA	docosahexaenoic acid
EPA	eicosapentaenoic acid
HDL	high-density lipoprotein
HFD	high-fat diet
LDL	low-density lipoprotein
MUFA	monounsaturated fatty acid
PUFA	polyunsaturated fatty acid
SFA	saturated fatty acid
TFA	*trans* fatty acid
iTFA	industrial-origin *trans* fatty acid
rTFA	ruminant-origin *trans* fatty acid
TG	triglyceride
